# A Parallel Human and Rat Investigation of the Interaction Between Descending and Spinal Modulatory Mechanisms

**DOI:** 10.1002/ejp.4775

**Published:** 2025-01-23

**Authors:** Anna Fieldwalker, Ryan Patel, Lucy Zhao, Mateusz W. Kucharczyk, Michael Mansfield, Kirsty Bannister

**Affiliations:** ^1^ Mroue Fateh Centre for Pain Management Great Ormond Street Hospital for Children Guildford Street London UK; ^2^ Wolfson Sensory, Pain and Regeneration Centre King's College London, Guy's Campus London UK; ^3^ School of Sport, Exercise and Rehabilitation Sciences, College of Life and Environmental Sciences University of Birmingham Birmingham UK; ^4^ Department of Life Sciences South Kensington Imperial College London London UK

**Keywords:** conditioned pain modulation, diffuse noxious inhibitory controls, dorsal horn, in vivo electrophysiology, pupillometry, quantitative sensory testing, temporal summation of pain, wide dynamic range, wind‐up

## Abstract

**Background:**

Healthy individuals demonstrate considerable heterogeneity upon dynamic quantitative sensory testing assessment of endogenous pain modulatory mechanisms. For those who stratify into a ‘pro‐nociceptive profile’ cohort, consisting of inefficient conditioned pain modulation (CPM) and elevated temporal summation of pain (TSP), the optimal approach for balancing the net output of pain modulatory processes towards anti‐nociception remains unresolved. In this translational healthy human and rat study, we examined whether descending modulation countered spinal amplification during concurrent application of a CPM and TSP paradigm alongside pupillometry since pontine activity was previously linked to functionality of endogenous pain modulatory mechanisms and pupil dilation.

**Methods:**

Perceptual (quantitative sensory testing) and spinal neuronal (in vivo electrophysiology) assessment was performed in healthy humans and rats respectively upon application of parallel CPM/diffuse noxious inhibitory controls (cuff algometry) and TSP/wind‐up (pinprick) paradigms alongside pupillometry.

**Results:**

In humans, repetitive pinprick stimulation produced TSP while concurrent application of a noxious conditioning stimulus did not affect pain ratings to a single pinprick stimulus, repetitive stimulation or the wind‐up ratio. In rats, repetitive pinprick produced neuronal wind‐up while concurrent application of a noxious conditioning stimulus inhibited neuronal responses to a single stimulus and repetitive stimulation but not the wind‐up ratio. For pupillometry experiments, dilatory responses did not increase during application of a TSP or CPM paradigm in humans, while reliable rat responses were not obtained.

**Conclusions:**

Under the conditions of our study, spinal amplification mechanisms surpassed descending inhibitory controls while pupillometry did not offer a reliable indicator of endogenous pain modulatory mechanism function.

**Significance:** In this translational healthy human and rat study, activity in descending inhibitory controls did not counter spinal amplification processes underpinned by wind up. Despite pupil dilation being previously linked to modulatory mechanisms, dilatory responses did not offer a reliable indicator of functionality. For pro‐nociceptive individuals exhibiting inefficient conditioned pain modulation and/or high temporal summation of pain, dampening faciliatory mechanisms rather than augmenting top‐down inhibitory processes may be a more effective pain‐relief strategy.

**Abbreviations**
BL: baseline
CPM: conditioned pain modulation
CS: conditioning stimulus
DNIC: diffuse noxious inhibitory controls
DPMS: descending pain modulatory system
LED: light‐emitting diode
NRS: numerical rating scale
PD: post‐discharge
PDT: pain detection threshold
PTT: pain tolerance threshold
RM: repeated measures
RS: repetitive stimulus
SS: single stimulus
TS: test stimulus
TSP: temporal summation of pain
VAS: visual analogue scale
WDR: wide dynamic range

## 1 Introduction

Nociceptive transmission is subject to modulation at multiple levels throughout the sensory neuraxis. The relative balance between signalling in naturally occurring ‘top‐down’ and ‘bottom‐up’ inhibitory and facilitatory pathways influences the overall experience of pain. Activity in such pathways may be assessed through the single or sequential application of dynamic quantitative sensory paradigms, for example to evoke conditioned pain modulation (CPM) (Granot et al. 2008; Patel et al. 2024; Vincenot et al. 2024) or temporal summation of pain (TSP) (Nie et al. 2005; Rolke et al. 2006; Vincenot et al. 2024), which may indirectly provide insight into putative central mechanisms of inhibitory and facilitatory nociceptive processing respectively. Since pro‐nociceptive sensory profiles, that is, inefficient CPM and/or high TSP, are recorded both in health (Coghill and Yarnitsky 2015; Gierthmuhlen et al. 2015; Kennedy et al. 2020; Petersen et al. 2024; Pud, Granovsky, and Yarnitsky 2009) and chronicity (McPhee, Vaegter, and Graven‐Nielsen 2020; Petersen et al. 2024; Rabey et al. 2015), understanding their interaction is necessary for the eventual refinement of analgesic approaches that tackle dysfunction in endogenous pain pathways. Therefore, the first aim of our study was to explore, in health, the relationship between inhibitory and facilitatory processes by concurrently administering paradigms to evoke CPM and TSP. Does activation of an inhibitory mechanism via CPM block TSP‐induced excitatory signalling?

There remains a desire for objective measures when correlating pain ratings with activity in naturally occurring pain processing pathways. Pupil area was previously measured as noxious somatosensory stimuli are known to induce a phasic dilatory response (Chapman et al. 1999). Crucially, pupil responses and pain ratings to heat stimuli were linked to key brainstem loci that govern modulatory outputs (Eisenach et al. 2017), supporting pupil dilation as a surrogate measure of activity in a sub‐population of locus coeruleus neurons (Joshi et al. 2016; McBurney‐Lin, Megemont, and Yang 2022). Thus, the second aim of our study was to investigate whether pupil dilatory responses provide a reliable indication of activity in endogenous pain modulatory processes.

Finally, we are interested in the translatability of paradigms to measure pain and nociception. Efforts to overcome limitations regarding the methods used to assess nociception and pain have led to parallel rodent and human studies investigating the predictive value of rodent models of sensitisation and modulation in the human setting (Cummins et al. 2020; O'Neill et al. 2015; Patel et al. 2024). Therefore, the third aim of our study was to build on these previous investigations by performing human psychophysics alongside rat neurophysiological recordings of wide dynamic range (WDR) neuronal activity using parallel techniques to induce TSP: is there concordance between neuronal and perceptual responses in humans and rats respectively? Following, we examined, using identical test paradigms, whether concurrent application of a conditioning stimulus to evoke activity in a descending inhibitory control pathway (representing a CPM paradigm in humans and diffuse noxious inhibitory controls in rats) and repetitive pinprick stimulation affected (1) TSP in healthy pain‐free subjects or (2) wind‐up of WDR neurones in healthy anaesthetised rats. Alongside psychophysical testing, pupillometry was performed.

## 2 Methods

### 2.1 Subjects for the Quantitative Sensory Testing Study

Forty healthy subjects were included in this study (30 female: 25.9 ± 1.3 years [range 18–46]; 10 male: 33.6 ± 3.9 years [range 19–52]). Information about the study was disseminated both via the King's College London fortnightly research volunteers email circular and within the department. Inclusion criteria specified that subjects should be between 18 and 70 years old, have a strong grasp of English, and be free of pain and medication (except contraception) on the day of testing. Exclusion criteria included acute or chronic pain conditions, dermatological issues at the site of testing, pregnancy, neurological disorders and musculoskeletal or inflammatory conditions. Subjects were requested not to consume alcohol in the 24 h period prior to testing and to avoid excessively strenuous exercise of the legs. All subjects provided informed consent prior to testing which took place within the Wolfson Sensory, Pain and Regeneration Centre, and were compensated £25 for their participation. The study was approved by King's College London Research Ethics Committee (Reference RESCM‐21/22‐22208) and performed according to the Helsinki Declaration (World Medical 2013).

### 2.2 Protocol for Quantitative Sensory Testing

#### 2.2.1 Pupillometry

**FIGURE 1:**
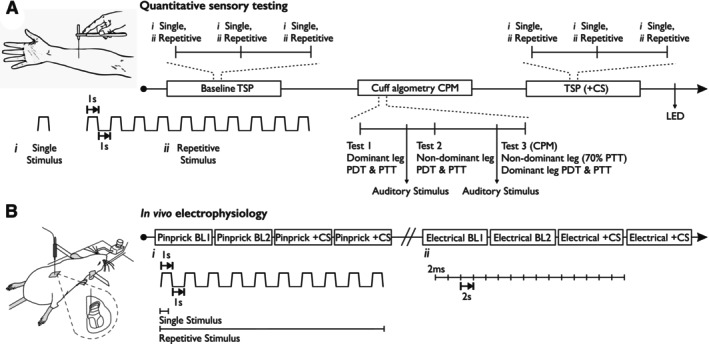
Schematics depicting experimental timeline for (A) quantitative sensory testing and pupillometry in humans and (B) in vivo electrophysiological recordings in rats. BL, baseline; CPM, conditioned pain modulation; CS, conditioning stimulus; LED, light‐emitting diode; PDT, pain detection threshold; PTT, pain tolerance threshold; TSP, temporal summation of pain.

Subjects attended a single session that lasted approximately 1.5 h (Figure 1A). At the beginning of the session, subjects were fitted with a helmet‐mounted infrared camera connected to a battery‐powered raspberry pi device. Videos were obtained in response to: a single pinprick stimulus (3), repetitive pinprick stimulation (3), cuff algometry—dominant leg (1), CPM (1), auditory stimulus (2), light control (1; data not shown). For repeated tests, data were averaged to produce a single value per subject. To determine a saliency response to a non‐somatosensory stimulus, at two points during testing the investigator triggered a surprise auditory stimulus triggered from a smart tablet device situated directly behind the subject. The first was a single low tone (‘buzzer’, less than 70 dB) lasting 1 s, and the second time two successive low tones lasting 1 s each. At the end of the session, to provide a physiological positive control, the subject's pupillary reflex was recorded. This was performed using blue light illumination lasting 1 s, shone into the ipsilateral pupil using a 375 nm wavelength 5 mm diameter light‐emitting diode (LED; NSPU510CS). Pupil diameter (ipsilateral to sensory testing) was recorded 10 s prior, during and for 10 s after the cessation of stimulation. Pupil area and blink detection were measured with MEYE (Figure S1), which is an open‐source web app that monitors pupil dynamics through deep learning (Mazziotti et al. 2021).

#### 2.2.2 Temporal Summation of Pain

TSP was assessed using a modified DFNS protocol (Rolke et al. 2006). A single 256 mN pinprick (1 s duration; MRC Systems GmbH, Germany) was applied to the dominant volar forearm and subjects were immediately asked to rate the painfulness of the stimulus on a numerical rating scale (NRS) from 0 (no pain at all) to 100 (the worst pain imaginable). Subjects were instructed that any sharp, stinging or pricking sensation was to be defined as being painful and given a rating value above 0. To measure temporal summation of pain, subjects experienced a subsequent series of 10 pinpricks, applied at a rate of 1 s on/1 s off within a 1 cm^2^ area of skin. After the end of stimulation, subjects immediately gave an overall pain rating for the 10‐pinprick series. In total three trials were performed 5 min apart on non‐overlapping areas of the forearm. The single stimulus and repetitive stimulus ratings were taken as the mean of three values. The wind‐up ratio was calculated as the mean repetitive stimulus rating divided by the mean single stimulus rating.

#### 2.2.3 Conditioned Pain Modulation

Cuff pressure pain sensitivity and CPM were determined using a computer controlled cuff algometry system (Nocitech CPAR, Aalborg University, Denmark) as previously described (Cummins et al. 2020; Patel et al. 2024). In brief, subjects experienced a slow increasing pressure ramp (1 kPa/s up to a maximum of 100 kPa) applied to the calf and were asked to rate pain intensity continuously on a visual analogue scale (VAS) using an electronic VAS device with a sliding bar which digitised to a 0–10 scale. The bar was anchored visually on the controller with ‘min’ and ‘max’, and verbally by the experimenters as ‘no pain at all’ and ‘the worst pain imaginable’. Pain detection threshold (PDT) was taken as the pressure (kPa) when a VAS score of 1 out of 10 was reached, and pain tolerance threshold (PTT) was taken when subjects self‐terminated the test indicating when they ‘cannot tolerate any more pressure’. For those who reached the maximum of 100 kPa, this was taken as their PTT. Firstly, the pressure ramp was applied to the dominant leg to determine PDT and PTT. The pressure ramp was then applied to the non‐dominant leg to determine PDT and PTT. To assess CPM, an increasing pressure ramp was applied to the dominant leg and PDT and PTT were determined whilst tonic pressure was applied to the non‐dominant leg (70% PTT). As per the recommended guidelines (Yarnitsky et al. 2015), the CPM effect was calculated as the baseline PDT minus the conditioned PDT measurements. This ensured that the presence of an inhibitory CPM effect was indicated by a negative value and a facilitatory CPM effect was indicated by a positive value.

#### 2.2.4 Temporal Summation of Pain With Conditioning Stimulus

Temporal summation of pain was re‐assessed with concurrent pressure (70% PTT) applied to the leg contralateral to the subject's dominant arm. For each test the pinprick stimuli were applied once the pressure ramp reached 70% PTT. Continuous tonic pressure was applied during the application of pinprick stimuli and was terminated after the end of the series. In total, three trials were performed 5 min apart. The single stimulus and repetitive stimulus ratings were taken as the mean of three values. The wind‐up ratio was calculated as the mean repetitive stimulus rating divided by the mean single stimulus rating.

### 2.3 Animals

Adult male Lister Hooded rats (nine in total; 8–10 weeks old, 250–300 g) were used for electrophysiological and pupillometry experiments (Charles River, UK). Animals were group housed (maximum of five) on a conventional 12:12 h light–dark cycle; food and water were available ad libitum. Temperature (20°C–22°C) and humidity (55%–65%) of holding rooms were closely regulated. Experimental design/analysis was conducted according to ARRIVE guidelines. All procedures described here were approved by an internal ethics panel and the UK Home Office (licence PABEF3413) under the Animals (Scientific Procedures) Act 1986 (SI 2012/3039).

### 2.4 Rat Pupillometry

Pupillometry recordings were performed prior to surgical preparation for electrophysiology under isoflurane anaesthesia (1%–1.25% *v/v* isoflurane). Animals were secured into a stereotaxic frame with eye retractors inserted. A Raspberry Pi computer (Raspberry Pi 3B+) coupled to infrared camera flanked by two IR diodes (Raspberry Pi noir camera module V2, to 30 fps at 720p resolution) was used for recordings. The system was wirelessly controlled using freeware Raspberry Pi Cam software (https://elinux.org/RPi‐Cam‐Web‐Interface) from a web browser on a PC within the same Wi‐Fi network. The camera was positioned on the ipsilateral eye and the baseline pupil dilation was set by dimming the ambient light to allow for dynamic range of responses. A set of six stimulations was delivered, as follows: (1) 40 kPa tonic cuff pressure applied to the right gastrocnemius muscle for 20 s, (2) repetitive pinprick stimulation of the left hindpaw (64 mN; 1 s on/1 s off) for 20 s, (3) repetitive pinprick stimulation of the left hindpaw with concurrent tonic cuff pressure applied to the right gastrocnemius muscle, (4) repetitive electrical stimulation of the left hindpaw (0.5 Hz, 2 ms square wave pulse, 3 mA) for 30 s, (5) 40 kPa tonic cuff pressure applied to the right gastrocnemius muscle for 30 s, (6) repetitive electrical stimulation of the left hindpaw with concurrent tonic cuff pressure applied to the right gastrocnemius muscle. Stimulation parameters and devices used were as described for the electrophysiology section below. Five minutes recovery was allowed between each stimulation and 10 s non‐stimulated baseline was collected for prior each stimulus. Video data were analysed with ImageJ (NIH, Bethesda, MD, USA). Briefly, following black and white conversion a line was drawn across the pupil's centre point extending to the side of the eye. Pixels brightness changes along the line was measured allowing to approximate dynamic pupil diameter (set grey threshold value, multi‐measure).

### 2.5 In Vivo Electrophysiology

Anaesthesia was initially induced with 3.5% *v/v* isoflurane delivered in 3:2 ratio of nitrous oxide and oxygen. Once areflexic, a tracheotomy/intubation was performed, and rats were subsequently maintained on 1.5% *v/v* isoflurane for the remainder of the experiment (approximately 3–4 h; core body temperature was maintained throughout with the use of a homeothermic blanket). Rats were secured in a stereotaxic frame and a laminectomy was performed to expose the L4‐L6 segments of the spinal cord; two spinal clamps were applied to stabilise the spinal column. Extracellular recordings were obtained from deep dorsal horn wide dynamic range (WDR) lamina V/VI neurones with receptive fields on the glabrous skin of the hind toes using 127 μm diameter 2 MΩ parylene‐coated tungsten electrodes (A‐M Systems, Sequim, WA). The search stimulus consisted of light tapping of the hind paw as the electrode was manually lowered. Neurones were characterised from depths relating to the deep dorsal horn laminae (726 ± 64 μm) (Watson et al. 2009), and once a single unit was isolated neurones were classified as WDR on the basis of sensitivity to dynamic brushing, noxious mechanical (60 g) and noxious heat stimulation (48°C) of the receptive field. Data were captured and analysed by a CED Micro1401 interface coupled to a computer with Spike2 v4 software (Cambridge Electronic Design, Cambridge, UK). The signal was amplified (×7500), bandpass filtered (low/high frequency cut‐off 0.5/2 kHz) and digitised at rate of 20 kHz.

The impact of diffuse noxious inhibitory controls (DNIC) on wind‐up was assessed by applying 40 kPa pressure via a neonatal cuff to the gastrocnemius muscle contralateral to the neuronal recording as previously described (Patel et al. 2024). Pinprick wind‐up was induced by repetitive application of a 64 mN pinprick stimulus (MRC Systems GmBH, Heidelberg, Germany) at a rate of 1 s on/1 s off repeated 10 times. The ‘single stimulus’ response was calculated as the number of spikes evoked by the first stimulus × 10 and represents the predicted non‐potentiated response. The ‘repetitive stimulus’ response represents the total neuronal response evoked by all 10 stimulations. Electrical wind‐up was subsequently determined; stimulation of WDR neurones was delivered transcutaneously via needles inserted into the receptive field. A train of 16 electrical stimuli (2 ms pulse width, 0.5 Hz) was applied at three times the threshold current for C‐fibre activation (via a NL800 current stimulus isolator, Digitimer Ltd., Welwyn Garden City, UK). Responses evoked by Aβ‐ (0–20 ms), Aδ‐ (20–90 ms) and C‐fibres (90–300 ms) were separated and quantified on the basis of latency. Neuronal responses occurring after the C‐fibre latency band were classed as post‐discharge (PD; 300–800 ms). The ‘single stimulus’ response was calculated as the number of spikes evoked by the first stimulus × 16. The ‘repetitive stimulus’ response was calculated as the total number of spikes evoked by all 16 stimuli. Wind‐up ratios were calculated as: WUR = repetitive stimulus response/single stimulus response. For both pinprick and electrical wind‐up experiments, two baseline trials were performed followed by two repeats in the presence of the conditioning stimulus (commencing 10 s prior to wind‐up and applied for the entire duration of stimulation) (Figure 1B). Trials were performed 10 min apart to avoid sensitisation of neurones. Data were averaged to produce control and conditioned responses.

### 2.6 Statistics

Statistical analyses were performed using SPSS v29 (IBM, Armonk, NY). All data collection and analyses were performed unblinded. Forty subjects were recruited for quantitative sensory testing, however seven were excluded from pupillometry analysis due to corrupted videos (1) and excessive blinking artefacts (6). For psychophysical measures the experimental unit was the individual subject; individual pain ratings were the within‐subject factor for comparisons. Data were assessed for normality (Kolmogorov–Smirnov) and then log transformed before comparison with a two‐way repeated measures ANOVA with Bonferroni post hoc for paired comparisons. The interaction (CPM × TSP) is reported in line with our aim of investigating whether activation of an inhibitory mechanism via CPM blocked TSP‐evoked excitatory signalling. Wind‐up ratios were compared with a Wilcoxon signed‐rank test. For pupillometry measures the experimental unit was the individual subject. To assess pupil dilatory responses, the maximum change in pupil area was compared with a Wilcoxon signed‐rank test in line with our aim to investigate whether pupil dilation provided a reliable indication of activity in endogenous pain modulatory processes. For electrophysiological measures the experimental unit was the individual rat; individual neuronal responses were the within‐subject factor for comparisons. Data were assessed for normality (Kolmogorov–Smirnov) and neuronal responses were compared with either a two‐tailed paired Student's *t*‐test or two‐way repeated measures (RM) ANOVA with Bonferroni post hoc for paired comparisons. Sphericity was tested using Mauchly's test; the Greenhouse–Geisser correction was applied if violated. Minimum group sizes were determined by a priori calculations using the following assumptions (*α* = 0.05, 1‐*β* = 0.8, *ε* = 1, effect size range *d* = 0.5–0.8). Effect sizes were determined from historical data sets. The animal group size balanced the need to ensure statistical robustness while adhering to the ‘3Rs’ (refine, reduce, replace; https://www.nc3rs.org.uk/the‐3rs). All data represent mean ± standard error (SEM). *p < 0.05, **p < 0.01, ***p < 0.001.

## 3 Results

### 3.1 In Healthy Humans, Applying a Noxious Conditioning Stimulus Does Not Suppress Temporal Summation of Pain in Dual CPM Inhibitors and TSP Facilitators

**FIGURE 2:**
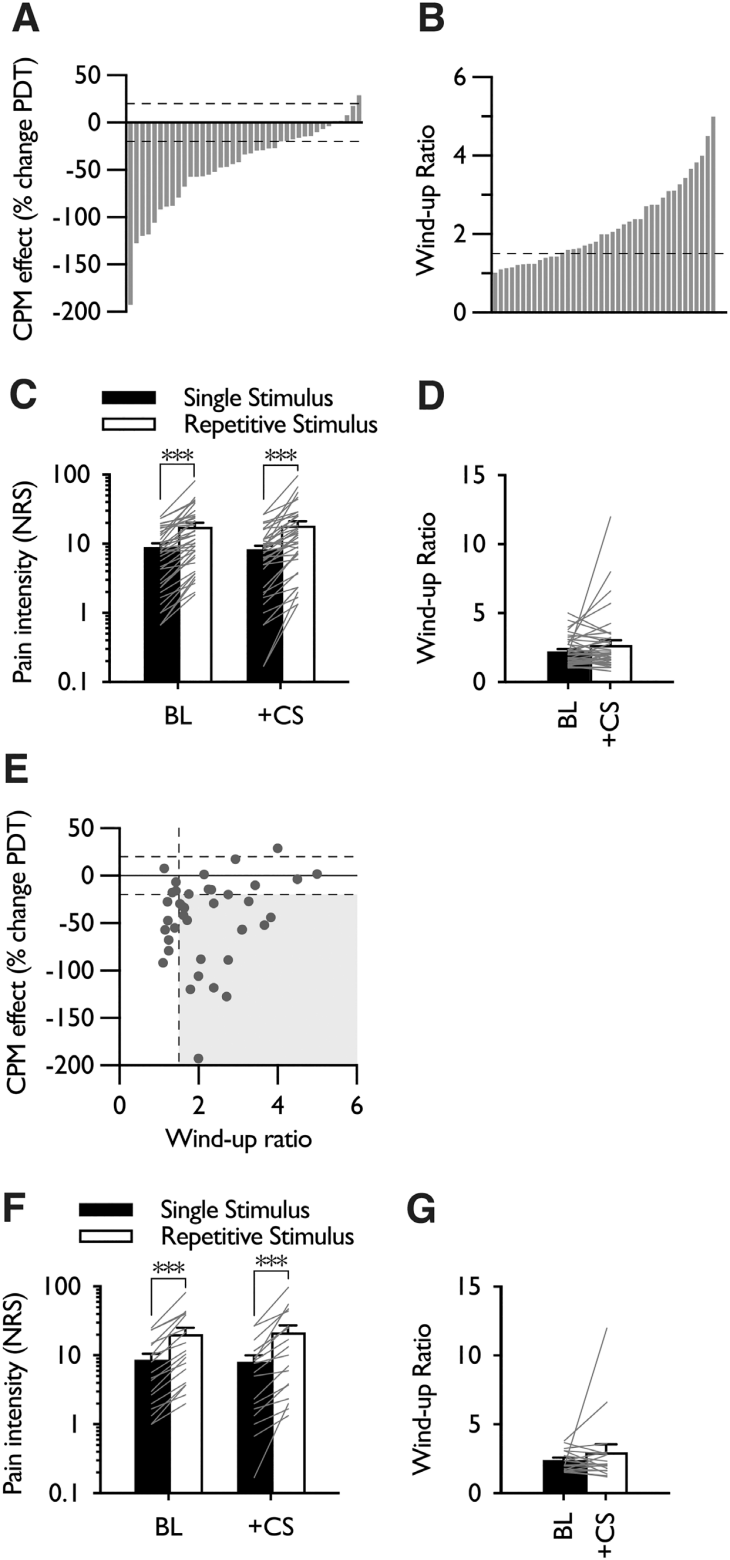
Applying a concurrent noxious conditioning stimulus did not affect temporal summation of pain to repetitive pinprick stimulation. (A) Range of CPM responses observed; columns represent individual responses. Dashed lines indicate a 20% change in pain detection threshold; positive values represent CPM facilitators and negative values represent CPM inhibitors. (B) Range of wind‐up ratios observed; columns represent individual responses. Dashed line indicates wind‐up ratio of 1.5. (C) Reported pain intensity to single pinprick stimulation and repetitive stimulation at baseline and during concurrent application of a conditioning stimulus in all subjects. (D) Temporal summation of pain expressed as a wind‐up ratio at baseline and during concurrent application of a conditioning stimulus in all subjects (*n* = 40). (E) Stratification of subjects based on an inhibitory CPM effect (> 20% change in PDT) and a wind‐up ratio greater than 1.5 (represented by the grey shaded area). (F) Reported pain intensity to single pinprick stimulation and repetitive stimulation at baseline and during concurrent application of a conditioning stimulus in the stratified cohort. (G) Temporal summation of pain expressed as a wind‐up ratio at baseline and during concurrent application of a conditioning stimulus in the stratified cohort (*n* = 18). Data represent mean ± SEM; ***p < 0.001. BL, baseline; CPM, conditioned pain modulation; CS, conditioning stimulus; NRS, numerical rating scale; PDT, pain detection threshold.

Based on a minimum 20% change of pressure pain detection threshold (PDT), 1/40 subjects were classified as CPM facilitators (i.e., their PDT decreased by > 20% when a conditioning stimulus was applied) and 25/40 as CPM inhibitors (i.e., their PDT increased by > 20% when a conditioning stimulus was applied) (Figure 2A), with TSP facilitators classified as having a wind‐up ratio of more than 1.5 following cut‐off values from previous studies (Graven‐Nielsen et al. 2015; Petersen et al. 2024; Vaegter and Graven‐Nielsen 2016) (Figure 2B). We were underpowered to assess sex differences in CPM efficiency and wind‐up ratios, however exploratory analysis revealed no difference in CPM effect (Male *n* = 10: −39.9% ± 9.1%; Female *n* = 30: −47.3% ± 9.1%. Mann–Whitney, *p* = 0.883) and no difference in wind‐up ratios (Male *n* = 10: 2.28 ± 0.28; Female *n* = 30: 2.21 ± 0.2. Mann–Whitney, *p* = 0.585).

Whole group analysis revealed that applying repetitive pinprick stimulation produced a temporal summation of pain effect and neither the single stimulus pain rating nor the repetitive stimulus pain rating was modulated by applying a noxious conditioning stimulus to the contralateral calf (two‐way RM ANOVA pinprick stimulus × CS interaction *F*_1,39_ = 1.866, *p* = 0.18) (Figure 2C). When the repetitive stimulus response was normalised to the single stimulus response the wind‐up ratio was unaffected by the application of a conditioning stimulus (Wilcoxon test, *p* = 0.353) (Figure 2D). The initial cohort was then stratified according to CPM responder status (increase in PDT > 20%) and a wind‐up ratio > 1.5 (Figure 2E). In this sub‐group of dual CPM inhibitors and TSP facilitators (18/40), we did not observe that applying a noxious conditioning stimulus modulated the single stimulus or repetitive stimulus pain rating (two‐way RM ANOVA pinprick stimulus × CS interaction *F*_1,17_ = 0.245, *p* = 0.627) (Figure 2F), nor the normalised wind‐up ratio (Wilcoxon test, *p* = 0.981) (Figure 2G).

### 3.2 In Healthy Humans, Pupil Dilatory Responses Did Not Provide a Reliable Indication of Activity in Endogenous Pain Modulatory Processes/Pain Intensity

**FIGURE 3:**
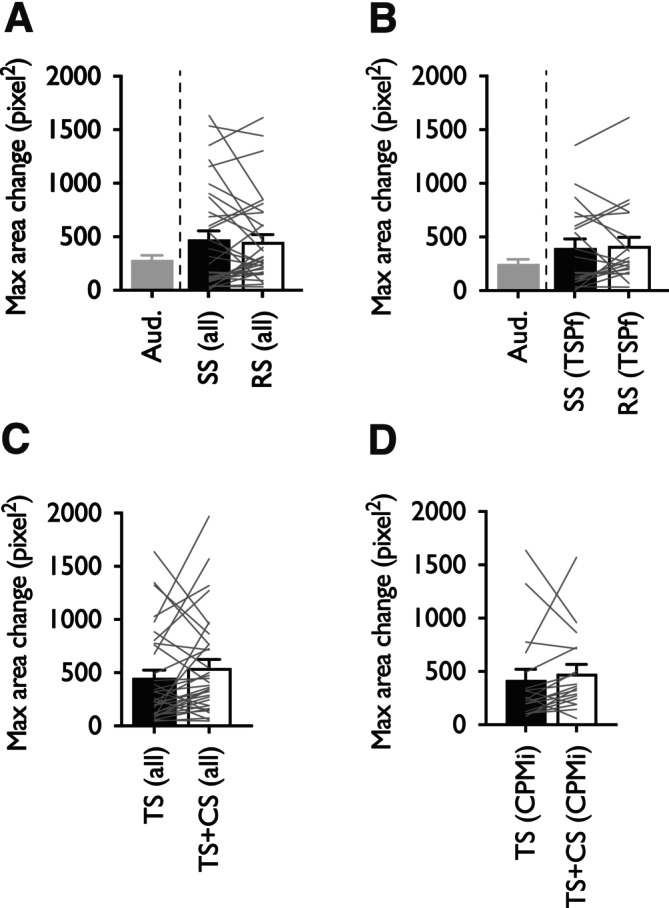
Pupil dilatory responses did not change during application of a temporal summation of pain or conditioned pain modulation paradigm. (A) Maximum pupil dilation in response to auditory tone (aud.), single pinprick stimulus and repetitive pinprick stimulus in all subjects (*n* = 33). (B) Maximum pupil dilation in response to auditory tone, single pinprick stimulus and repetitive pinprick stimulus in temporal summation of pain facilitators (*n* = 20; wind‐up ratio > 1.5). (C) Maximum pupil dilation in all subjects (*n* = 33) in response to increasing pressure applied to the test leg in the absence (TS) and presence (TS + CS) of a conditioning stimulus. (D) Maximum pupil dilation in conditioned pain modulation inhibitors (*n* = 18; > 20% increase in PDT) in response to increasing pressure applied to the test leg in the absence (TS) and presence (TS + CS) of a conditioning stimulus. Data represent mean ± SEM. CPMi, conditioned pain modulation inhibitors; CS, conditioning stimulus; RS, repetitive stimulus; SS, single stimulus; TS, test stimulus; TSPf, temporal summation of pain facilitators.

Pupillometry was conducted concurrently to quantitative sensory testing. Whole group analysis revealed maximum pupil dilatory responses during repetitive pinprick stimulation did not differ from responses to a single pinprick stimulus (Wilcoxon test, *p* = 0.755) (Figure 3A). The initial cohort was stratified according to the wind‐up ratio (> 1.5) and likewise maximum pupil dilatory responses during repetitive pinprick stimulation did not differ from responses to a single pinprick stimulus (Wilcoxon test, *p* = 0.526) (Figure 3B). During application of a CPM paradigm, whole group analysis revealed maximum pupil dilatory responses to the test plus conditioning stimulus did not differ from responses to the test stimulus alone (Wilcoxon test, *p* = 0.138) (Figure 3C). The initial cohort was stratified according to CPM efficiency (increase in PDT > 20%) and likewise maximum pupil dilatory responses to the test plus conditioning stimulus did not differ from responses to the test stimulus alone (Wilcoxon test, *p* = 0.184) (Figure 3D).

### 3.3 In Healthy Rats, Activation of Diffuse Noxious Inhibitory Controls Reduced Basal Neuronal Excitability but Not Pinprick‐ Or Electrically‐Induced Wind‐Up

**FIGURE 4:**
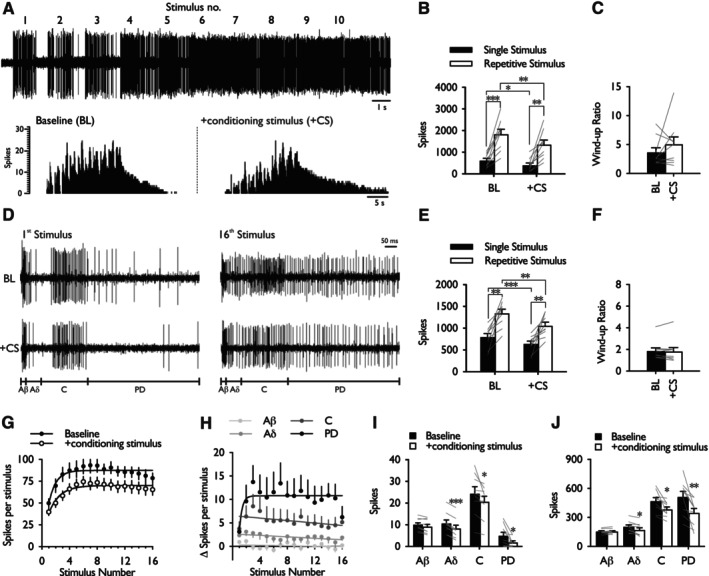
Activation of diffuse noxious inhibitory controls inhibited Aδ‐ and C‐fibre evoked activity but not spinal neuronal wind‐up. (A) Representative neurogram and histogram traces of a single unit response to repetitive 64 mN pinprick stimulation. (B) Effect of activating DNIC on single stimulus and repetitive pinprick evoked neuronal response. (C) Effect of activating DNIC on pinprick wind‐up ratio. (D) Representative neurogram traces of a single unit response to repetitive electrical stimulation. (E) Effect of activating DNIC on single stimulus and repetitive electrically evoked neuronal response. (F) Effect of activating DNIC on electrical wind‐up ratio. (G) Wind‐up of deep dorsal horn neurones expressed as mean spikes per stimulus number. (H) Difference in evoked spikes between baseline and conditioned trials expressed as mean spikes per stimulus number separated according to latency. (I) Effect of DNIC on afferent evoked activity after the first electrically evoked response. (J) Effect of DNIC on total neuronal events evoked by the 16 stimulus train. Data represent mean ± SEM; *n* = 9. *p < 0.05, **p < 0.01, ***p < 0.001. BL, baseline; CS, conditioning stimulus; PD, post‐discharge.

Electrophysiological recordings were obtained from dorsal horn lamina V/VI wide dynamic range neurones. Application of repetitive pinprick stimulation to the centre of the peripheral receptive field produced a pronounced wind‐up of the evoked response (Figure 4A). DNIC were effectively activated by the conditioning stimulus (cuff pressure applied to the contralateral leg) as evidenced by a decrease in the single stimulus (*d* = −0.703) and repetitive stimulus (*d* = −0.702) responses (two‐way RM ANOVA pinprick stimulus × DNIC interaction *F*_1,8_ = 14.53, *p* = 0.005) (Figure 4A,B). However, when the repetitive stimulus response was normalised to the single stimulus response the wind‐up ratio was unaffected by activating DNIC (Wilcoxon test, *p* = 0.515) (Figure 4C).

The effect of activating DNIC on electrically evoked wind‐up was subsequently examined as means to ensure that the effect of pinprick‐induced wind‐up and subsequent interaction with DNIC was not modality dependent (Figure 4D,E). Electrically‐induced wind‐up was modulated in a similar manner to pinprick‐induced wind‐up as the single stimulus (*d* = −0.724) and repetitive stimulus (*d* = −1.053) responses were both inhibited (two‐way RM ANOVA pinprick stimulus × DNIC interaction *F*_1,8_ = 5.552, *p* = 0.046) (Figure 4D,E), but when normalised to the single stimulus response the wind‐up ratio was unaffected by activating DNIC (Wilcoxon test, *p* = 0.407) (Figure 4F). Repetitive electrical stimulation produced a biphasic wind‐up response with an accelerating phase followed by a plateau phase. Activating DNIC did not alter the kinetics of wind‐up and the rate constants in the first phase were comparable to the baseline response (*k*_BL_ = 0.9656, *k*_+CS_ = 0.6414; sum‐of‐squares *F*‐test, *p* = 0.228) (Figure 4G). The decrease in the evoked response was examined in greater detail on a stimulation‐by‐stimulation basis and according to response latency. The magnitude of inhibition of Aδ‐ and C‐evoked activity appeared consistent across the entire stimulus train (one‐way RM ANOVA, Aδ: *F*_15,120_ = 0.833, *p* = 0.584; C: *F*_15,120_ = 0.773, *p* = 0.705), whereas weak evidence was found to support a biphasic modulation of post‐discharge events (1‐way RM ANOVA, *F*_15,120_ = 1.697, *p* = 0.06) (Figure 4H). DNIC exerted a differential inhibitory effect on afferent evoked activity in response to the first electrical stimulus; Aβ‐evoked activity was unaffected whereas Aδ‐, C‐ and post‐discharge (PD) events decreased (paired Student's *t*‐test, Aβ: *p* = 0.076, *t* = 2.036; Aδ: *p* = 0.00013, *t* = 6.791; C: *p* = 0.03, *t* = 2.632; PD: *p* = 0.046, *t* = 2.365, *df* = 8) (Figure 4I). At the end of the repetitive stimulus train cumulative Aβ‐evoked activity remained unaffected whereas Aδ‐, C‐ and post‐discharge events decreased (paired Student's *t*‐test, Aβ: *p* = 0.766, *t* = 0.308; Aδ: *p* = 0.013, *t* = 3.171; C: *p* = 0.011, *t* = 3.267; PD: *p* = 0.006, *t* = 3.703, *df* = 8) (Figure 4J).

### 3.4 Rat Pupillary Responses Failed to Be Reliably Measured Under the Experimental Conditions

Pupillometry recordings were obtained as standalone but during the electrophysiology experiments. None of the stimulation paradigms employed produced visible pupil response (Figure S2).

## 4 Discussion

In this translational study, wherein parallel human and rat protocols were applied, we investigated the interaction between perceptual responses in humans and neurophysiological responses in rats upon repetitive cutaneous stimulation in the absence and presence of a heterotopic noxious conditioning stimulus. We found that activation of the descending pain modulation system (DPMS) using a CPM (humans) or DNIC (rats) paradigm did not affect TSP or neuronal wind‐up respectively that is, activation of an inhibitory mechanism via CPM or DNIC did not block excitatory signalling under the conditions of our study.

The major advantage of our experimental design was that we could define the input–output relationship between peripheral stimuli and psychophysical responses in humans, and spinal output in rats upon application of identical experimental paradigms. We found concordance between human psychophysical and rat spinal neuronal responses supporting their translational validity, that is, we could correlate human perceptual and rat neurophysiological responses. The ability to align neuronal correlates with commonly applied psychophysical techniques enhances confidence in observed effect sizes while ultimately illuminating mechanisms and/or risk factors of disease. While application of a conditioning stimulus concurrent to single pinprick stimulation inhibited spinal neuronal activity but not human perceptual responses, we note that the sensitivity to detect a subtle effect is higher with direct neuronal recordings.

In humans, all TSP responses bar one facilitated upon repetitive stimulation of the periphery above a critical rate, and CPM responses were variable. These data are in line with previous findings that highlighted the heterogeneity by which an individual responds to the CPM paradigm (Coghill and Yarnitsky 2015; Edwards et al. 2003; Ge, Madeleine, and Arendt‐Nielsen 2004; Gierthmuhlen et al. 2015; Imai et al. 2016; Kennedy et al. 2020; Patel et al. 2024; Pud, Granovsky, and Yarnitsky 2009). Due to the size of our cohort combined with the number of potential responder groups (i.e., facilitator, inhibitor or non‐responder), we were not powered to observe whether stratifying into separate groups revealed any impact of application of a conditioning stimulus on TSP. However, the existing analyses observing whole group and dual CPM inhibitor/TSP facilitator data suggest that responder status does not modify the effect of a conditioning stimulus on wind‐up ratio.

Previous studies investigating the interaction between CPM and TSP have produced conflicting results (Table 1) with reports of a decrease (Graven‐Nielsen et al. 2015; Haack et al. 2012; Horn‐Hofmann et al. 2018; Staud et al. 2003; Streff, Michaux, and Anton 2011), increase (Holden et al. 2019), or no effect (Cathcart et al. 2010; Garrett et al. 2013; Horn‐Hofmann et al. 2018; Lautenbacher, Kunz, and Burkhardt 2008; Oono et al. 2013; Streff, Michaux, and Anton 2011) on wind‐up. These divergent results could be due to the nature of the stimuli applied (i.e., cutaneous vs. deep inputs or stimulus modality) or the activation of additional modulatory mechanisms by different protocols. Unfortunately, an identical research question does not automatically incorporate an identical protocol *modus operandi*, limiting interpretation. A recent systematic review engaged with this point, outlining the deleterious impact of paradigm heterogeneity on TSP investigation in certain chronic pain states (Huisman et al. 2024). In addition, our understanding of the impact of peripheral nervous system activity on the expression of modulatory controls is lacking. Stimulus modality will alter input to, and thus the recorded output of, descending and spinal circuits. Our finding that spinal amplification mechanisms surpass inhibitory controls is relevant only for the modalities applied in our study. In the future, exploring these effects using different protocols is physiologically relevant since paradigms to elicit CPM are not always applied in the context of modulating an additional ‘experimental’ (i.e., pinprick evoked wind‐up) pain. How ‘high’ does the spinal amplification process need to be before inhibitory controls can no longer modulate the perceived effect?

**TABLE 1:** Summary of modulatory effects of a heterotopic noxious conditioning stimulus on psychophysical measures of temporal summation of pain in healthy subjects.

Study	*n*	TS frequency	TS modality	CS modality	Effect of CS on SS pain rating	Effect of CS on RS pain rating	Effect of CS on WUR
Streff, Michaux, and Anton (2011)	24	1 Hz	Mechanical impact	Heat	No change	Decrease	Decrease
Streff, Michaux, and Anton (2011)	24	1 Hz	Mechanical impact	Pressure	No change	Decrease	Decrease
Current study	40	1 s on/1 s off	Punctate mechanical	Pressure	No change	No change	No change
Oono et al. (2013)	24	1 Hz	Punctate mechanical	Pressure	Not reported	Not reported	No change
Garrett et al. (2013)	30	0.5 Hz	Punctate mechanical	Cold	No change	No change	No change
Lautenbacher, Kunz, and Burkhardt (2008)	40	0.5 Hz	Pressure	Heat	Decrease	Decrease	No change
Horn‐Hofmann et al. (2018)	35	0.5 Hz	Pressure	Heat	Decrease	Decrease	No change
Cathcart et al. (2010)	25	1 s on/1 s off	Pressure	Pressure	Decrease	Decrease	No change
Holden et al. (2019)	20	1 s on/1 s off	Pressure	Pressure	Decrease	No change	Increase
Graven‐Nielsen et al. (2015)	68 + 68	2 s on/1 s off	Pressure	Cold	Not reported	Not reported	Decrease (F), no change (M)
Horn‐Hofmann et al. (2018)	35	0.5 Hz	Heat	Heat	No change	Decrease	Decrease
Haack et al. (2012)	17	0.4 Hz	Heat	Heat	Not reported	Not reported	Decrease
Staud et al. (2003)	11 + 22	0.5 Hz	Heat	Heat	Not reported	Not reported	Decrease (M), no change (F)
Streff, Michaux, and Anton (2011)	24	1 s on/1 s off	Heat	Heat	Decrease	Decrease	Decrease
Streff, Michaux, and Anton (2011)	24	1 s on/1 s off	Heat	Pressure	Decrease	Decrease	No change

Abbreviations: CS, conditioning stimulus; F, female; M, male; RS, repetitive stimulus; SS, single stimulus; TS, test stimulus; WUR, wind‐up ratio.

Historically spinal neuronal wind‐up has been assessed with electrical stimulation. The translational relevance to TSP has been questioned with some arguing that the synchronised supra‐threshold nature of electrical stimulation recruits distinct amplification mechanisms compared to natural stimulation which inevitably evokes asynchronous activity (Herrero, Laird, and Lopez‐Garcia 2000). Here we demonstrated that applying a modified DFNS temporal summation of pain paradigm produced spinal neuronal wind‐up in rats. Moreover, these neurones also exhibited electrically‐induced wind‐up, and activation of DNIC modulated repetitive electrically and mechanically evoked activity in a similar manner. DNIC inhibited the single and repetitive stimulus responses to a similar extent resulting in no overall effect on the wind‐up ratio. Activation of DNIC appears independent of stimulus modality and requires heterotopic activation of Aδ‐ and C‐fibres (Bouhassira, Le Bars, and Villanueva 1987; Le Bars, Dickenson, and Besson 1979), and in the descending arm of the circuit we observed that DNIC inhibited Aδ‐ and C‐fibre evoked activity during electrical wind‐up consistent with previous reports (Schouenborg and Dickenson 1985). The effect of the conditioning stimulus was to inhibit Aδ‐ and C‐fibre evoked activity by a constant number of spikes independent of wind‐up phase or firing frequency implicating a presynaptic inhibitory mechanism, either via inhibition of primary afferent terminals or interneurons. This circles back to the discussion on the importance of modality, where the underpinning mechanism(s) of the observed net effect will differ according to the nature of peripheral stimulation. It also brings to the fore that even the fundamental nature of DNIC in terms of a presynaptic primary afferent versus post synaptic spinal effect remains to be fully elucidated.

Finally, in terms of the translational comparison of CPM and DNIC, it is important to recall that DNIC underlies only a component of the CPM response (Nahman‐Averbuch et al. 2024). Indeed, our finding that DNIC significantly decreased neuronal responses evoked by single or repetitive responses while CPM produced no changes on the pain ratings of single and repetitive stimuli serves as a reminder that DNIC and CPM are distinct phenomena. This is also evident when considering that DNIC expression is uniform in pre‐clinical anaesthetised animal preparations (Bannister et al. 2015; Kucharczyk, Di Domenico, and Bannister 2023; Patel et al. 2024) while the response to application of a paradigm to evoke CPM in wakeful humans is varied (Edwards et al. 2003; Kennedy et al. 2020; Pud, Granovsky, and Yarnitsky 2009; Rabey et al. 2015).

Locus coeruleus activity and dynamic pupil responses are associated with somatosensory evoked responses (Larsen and Waters 2018). The expression of DNIC is also reliant on pontine nuclei and DNIC is likely one of multiple mechanisms recruited during CPM (Kucharczyk, Di Domenico, and Bannister 2022, 2023; Nahman‐Averbuch et al. 2024), thus pupil dilatory responses may correlate with the CPM effect. When performing pupillometry concurrent to application of a temporal summation of pain or conditioned pain modulation paradigm, pupil dilation did not offer a reliable indicator of activity in endogenous pain modulatory processes. Although previous studies have described phasic dilatory responses to noxious stimulation in an intensity‐dependent manner, the reliability of the dilatory response increases with higher stimulus intensities and may explain the lack of correlation we observed (Eisenach et al. 2017). Indeed, while people with higher pain ratings were suggested as having a higher variance of pupillary response, the pupillary response itself was previously shown not to correlate with pain ratings, suggestive of pupil diameter and numerical pain rating as capturing different aspects of the pain experience (Krafthofer et al. 2024). We did not observe an increase in pupil dilation during the conditioned pain modulation test either, in line with previous findings (Eisenach et al. 2017). With regards to the pupillometry application in rats, we observed no changes in pupil diameter upon application of our paradigms due to the confounds of anaesthesia. The conditions applied prior to in vivo electrophysiology preparation are important limitation and consideration factors that should be highlighted for researchers in this field of expertise.

A further limitation is that while our human subjects were both male and female, we were not powered to detect sex‐based differences (and note that sex differences was not a factor of investigation for our study). Meanwhile, only male animals were used. While animal sex, and even strain, have previously been shown to impact behavioural DNIC outcomes (Da Silva et al. 2020), our experiments were performed in anaesthetised rats where an impact of sex has not previously been reported for the neurophysiological outcomes we were recording (Bannister et al. 2015; Trendafilova et al. 2022; Valiente 2024). As such, we are confident that our use of only male rats did not impact the results.

In conclusion, targeting endogenous excitatory or inhibitory mechanisms as a way of balancing the net output of naturally occurring pain modulatory mechanisms towards anti‐nociception is only possible and/or worthwhile if we (a) understand the influence of converging spinal and brain networks on pain processing and (b) can measure the output of those networks in a standardised and reproducible manner. In overcoming these shortfalls, the application of studies that expose the net output of the DPMS could be beneficial in so‐called therapeutic ‘risk analyses’, which may reveal who has a pre‐loading vulnerability to developing a chronic pain state and who is most likely to benefit from specific DPMS‐targeted interventions.

## Author Contributions

All authors contributed to the conception and design of the study and data interpretation. K.B. and R.P. drafted the manuscript and, following critical review and contribution from each author, finalised manuscript preparation for publication. A.F., R.P., and M.M. performed experiments while all authors contributed to data analysis.

## Conflicts of Interest

The authors declare no conflicts of interest.

## Supporting information


Figure S1.

Figure S2.

## Data Availability

All data are available upon request to the corresponding author.
