# Supplementary material for: A Parallel Human and Rat Investigation of the Interaction Between Descending and Spinal Modulatory Mechanisms
Source: Eur J Pain. 2025 Jan 23;29(3):e4775. doi: 10.1002/ejp.4775 (PMC11758248; doi:10.1002/ejp.4775)
Supplement: Supplementary file 1 — Figure S1. [file EJP-29-0-s001.pdf]

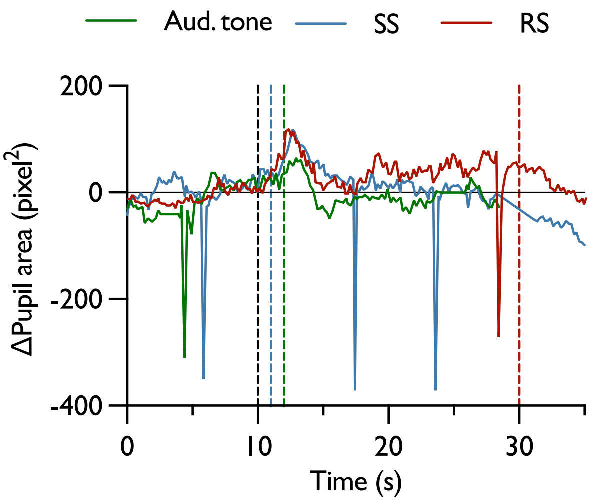

Supplementary Figure S1. Representative pupil dilatory response of an individual subject during application of a temporal summation of pain paradigm. The change in pupil area was normalised to the area at 10 s i.e. at the start of stimulation. Dashed vertical lines denote the start (black) and end of stimulation periods (green – auditory tone (aud.), blue - single 256 mN pinprick stimulus (SS), red – repetitive 256 mN stimulus (RS)).
