# Supplementary material for: A Parallel Human and Rat Investigation of the Interaction Between Descending and Spinal Modulatory Mechanisms
Source: Eur J Pain. 2025 Jan 23;29(3):e4775. doi: 10.1002/ejp.4775 (PMC11758248; doi:10.1002/ejp.4775)
Supplement: Supplementary file 2 — Figure S2. [file EJP-29-0-s002.pdf]

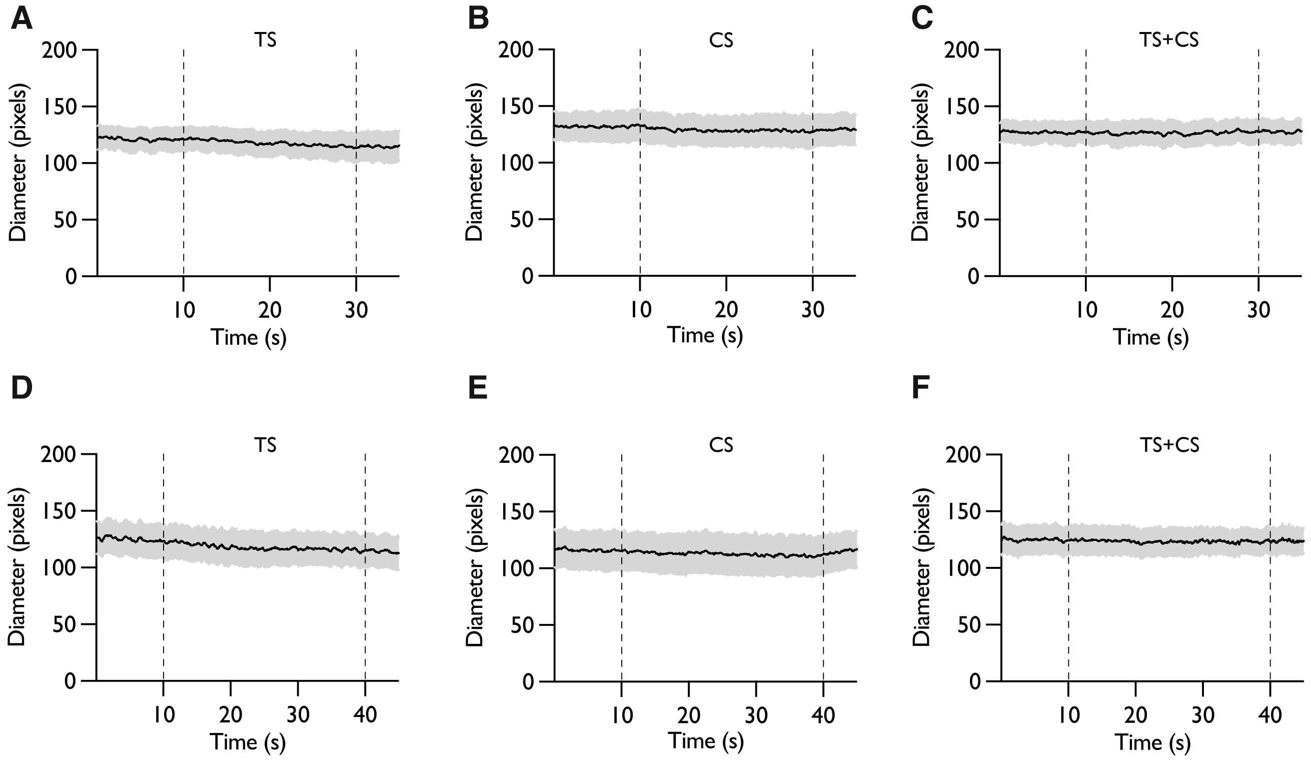

Supplementary Figure S2. Rat pupil dilatory responses did not change during application of a wind-up or diffuse noxious inhibitory controls paradigm. (A) Left pupil diameter in response to repetitive pinprick stimulation (TS) of the left hindpaw (64 mN; 1 s on/1 s off); dashed vertical lines denote stimulation period. (B) Left pupil diameter in response to 40 kPa tonic cuff pressure (CS; 20 s) applied to the right gastrocnemius muscle. (C) Left pupil diameter in response to repetitive pinprick stimulation of the left hindpaw with concurrent tonic cuff pressure applied to the right gastrocnemius muscle (TS+CS). (D) Left pupil diameter in response to repetitive electrical stimulation (TS) of the left hindpaw (0.5 Hz, 2 ms pulse, 3 mA). (E) Left pupil diameter in response to 40 kPa tonic cuff pressure (CS; 30 s) applied to the right gastrocnemius muscle. (F) Left pupil diameter in response to repetitive electrical stimulation of the left hindpaw with concurrent tonic cuff pressure applied to the right gastrocnemius muscle (TS+CS). Data represent mean ± SEM; *n*=6. CS – conditioning stimulus, TS – test stimulus.
